# Recurrent intussusception caused by giant ileal gastric heterotopia in a patient with ileocecectomy: A rare case report and literature review

**DOI:** 10.1016/j.ijscr.2025.112006

**Published:** 2025-10-01

**Authors:** Foolad Eghbali, Mohammadsadra Shamohammadi, Asghar Arabhosseini, Parisa Pooyan, Nassir Mohseni jam, Mohammadreza Javaherian

**Affiliations:** a Department of Surgery, Rasool-E Akram Hospital, School of Medicine, Iran University of Medical Sciences, Tehran, Iran; b Gastrointestinal and Liver Diseases Research Center, Iran University of Medical Sciences, Tehran, Iran; c Shahid Beheshti University of Medical Sciences, Tehran, Iran

**Keywords:** Intussusception, Gastric heterotopia, Recurrent intussusception, Ileal polyp, Intestinal heterotopia, Case report

## Abstract

**Introduction:**

Adult intussusception is rare, accounting for approximately 5 % of all intussusceptions and 1–5 % of bowel obstructions in adults. Gastric heterotopia of the small intestine is an uncommon lead point. This case underscores the need to consider gastric heterotopia in recurrent presentations. Recognizing this entity can facilitate prompt diagnosis, guide selection between selective reduction and en bloc resection, and expedite definitive management.

**Presentation of case:**

A 31-year-old woman with prior ileocecectomy for intussusception presented with worsening colicky right lower-quadrant pain. CT and colonoscopy showed ileo-ileal intussusception. Controlled pneumatic reduction revealed a polypoid ileal mass; biopsies indicated gastric heterotopia. She underwent laparoscopic segmental ileal resection (10 cm) with side-to-side extracorporeal anastomosis. Histology confirmed polypoid gastric heterotopia with clear margins. Recovery was uneventful with complete symptom resolution.

**Discussion:**

Adult intussusception is uncommon and typically arises from a pathologic lead point. Ileal gastric heterotopia is a rare benign cause with potential for recurrence. Cross-sectional imaging, often complemented by endoscopy, helps characterize the lesion and assess for ischemia. When findings indicate a benign intraluminal process without ischemia or suspected malignancy, selective reduction can aid localization and facilitate a limited, minimally invasive resection; when malignancy is suspected, en bloc resection without reduction is preferred. Definitive management is segmental resection with histopathologic confirmation.

**Conclusion:**

Ileal gastric heterotopia is an exceptionally rare cause of recurrent adult intussusception, including after prior bowel resection. Surgical resection remains definitive and yields favorable outcomes.

## Highlights

• Recurrent adult intussusception caused by giant ileal gastric heterotopia is extremely rare
• CT and colonoscopy were pivotal in diagnosing intussusception and identifying gastric heterotopia.
• Laparoscopic segmental resection of the involved ileal segment achieved complete removal with clear margins

## 1 Introduction

Adult intussusception is an uncommon condition, accounting for approximately 5 % of all intussusceptions and 1–5 % of bowel obstruction in adults with an incidence of 2–3 per 1,000,000 annually [1]. Unlike in children, where intussusception is typically idiopathic, adult cases generally have an identifiable lead point, with neoplasms being the most common etiology [1,2]. The pathophysiology involves telescoping of a proximal segment of bowel (intussusceptum) into an adjacent distal segment (intussuscipiens), leading to obstruction and possible ischemia [3]. Clinical presentation in adults is often nonspecific and chronic, characterized by intermittent abdominal pain, nausea, vomiting, and altered bowel habits, posing a diagnostic challenge and often delaying treatment [2]. Gastric heterotopia, defined as the existence of gastric mucosa at an abnormal site without connection to the stomach, is a rare cause of intussusception, particularly in the ileum [4,5]. The polypoid nature of heterotopic tissue can interfere with normal peristalsis and create a mechanical lead point [6].

We present a rare case of recurrent intussusception due to giant ileal gastric heterotopia in a patient with previous ileocecectomy and further examine the literature on this uncommonly seen clinical phenomenon. This case emphasizes the need to maintain a high index of suspicion for rare etiologies in recurrent presentations and highlights successful surgical intervention. This report follows the SCARE 2025 criteria for case reports [7].

## 2 Case presentation

A 31-year-old woman patient presented to our surgical outpatient department with a 7-day history of progressively worsening colicky abdominal pain predominantly in the right lower quadrant (RLQ) and hypogastric region. Pain was colicky, lasting 20–30 min per episode with intermittent relief. Associated symptoms included mild nausea without vomiting, loss of appetite, and subjective abdominal distension. She denied having any fever, hematochezia, or melena. The patient reported prior similar, self-limiting episodes of abdominal pain over the past three months. The vital signs were normal. Her surgical history included an ileocecectomy performed for intussusception, with benign inflammation as the lead point and an uncomplicated postoperative course. There was no significant family history of gastrointestinal diseases. Physical examination revealed abdominal distention and tenderness in the right lower quadrant without signs of peritoneal irritation. Hematologic and complete metabolic panel was within normal limits.

**Fig. 1:**
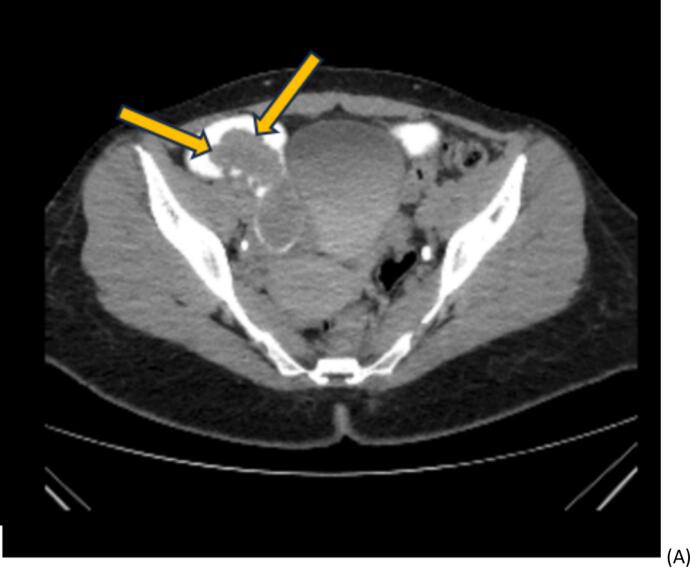
Contrast-enhanced CT scans demonstrating a tumor in the right quadrant of the abdomen with the characteristic bowel-within-bowel configuration suggestive of intussusception (A: axial view; B: coronal view, arrows).

**Fig. 2:**
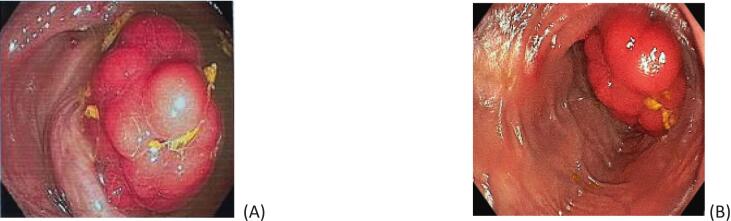
**(A)** Pre-reduction colonoscopy revealed a pedunculated, localized polyp in the terminal ileum at the 100-cm level, with a large, fungating mass identified as the lead point of intussusception. **(B)** Colonoscopy after reduction.

Ultrasonography and a contrast-enhancement abdominopelvic CT scan confirmed an ileo-ileal intussusception with partial bowel obstruction (Fig. 1. Within the intussusceptum, a 3 × 5 cm hypodense intraluminal mass likely representing a lead point was seen. There were no signs of bowel ischemia, including pneumatosis intestinalis, portal venous gas, or free intraperitoneal air were observed. The colonoscopy showed normal mucosa up to the level of ileocolic anastomosis. Upon advancing the colonoscope into the neoterminal ileum, an ileo-ileal intussusception was visualized. Adult enteric intussusceptions are benign in the majority of cases, with lipomas accounting for about 27 %, while malignant tumors are found in only 20 % of enteric cases. [[8], [9], [10]] Given our CT showing no ischemia and an endoscopically smooth intraluminal lesion, we elected a controlled endoscopic pneumatic reduction to obtain targeted biopsies and to guide a limited laparoscopic resection. Post-reduction endoscopy identified 5-cm polypoid ileal lesion ∼100 cm proximal to the anastomosis with mild superficial ulceration; targeted biopsies were obtained. (Fig. 2). Biopsy of the polyp and overlying ileal mucosa was performed.

**Fig. 3:**
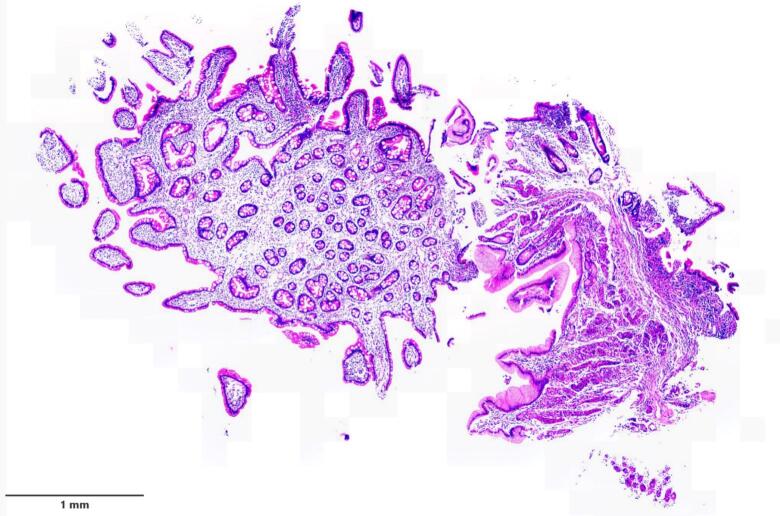
Heterotopic gastric mucosa, Higher power view of the heterotopic gastric mucosa, ileal mucosa biopsy.

**Fig. 4:**
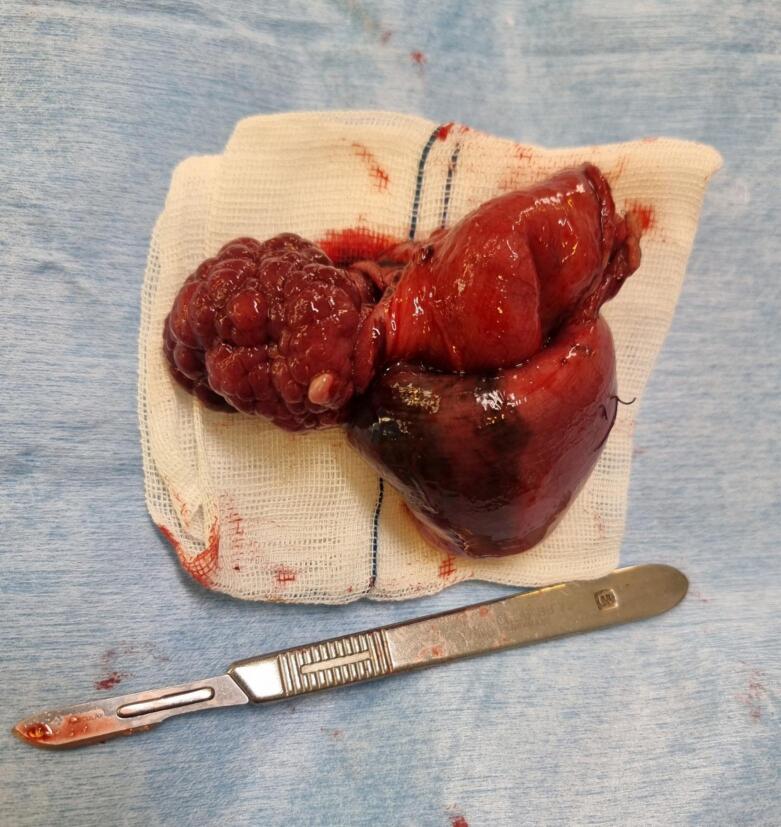
Resection of ileum contained fungating mass.

**Fig. 5:**
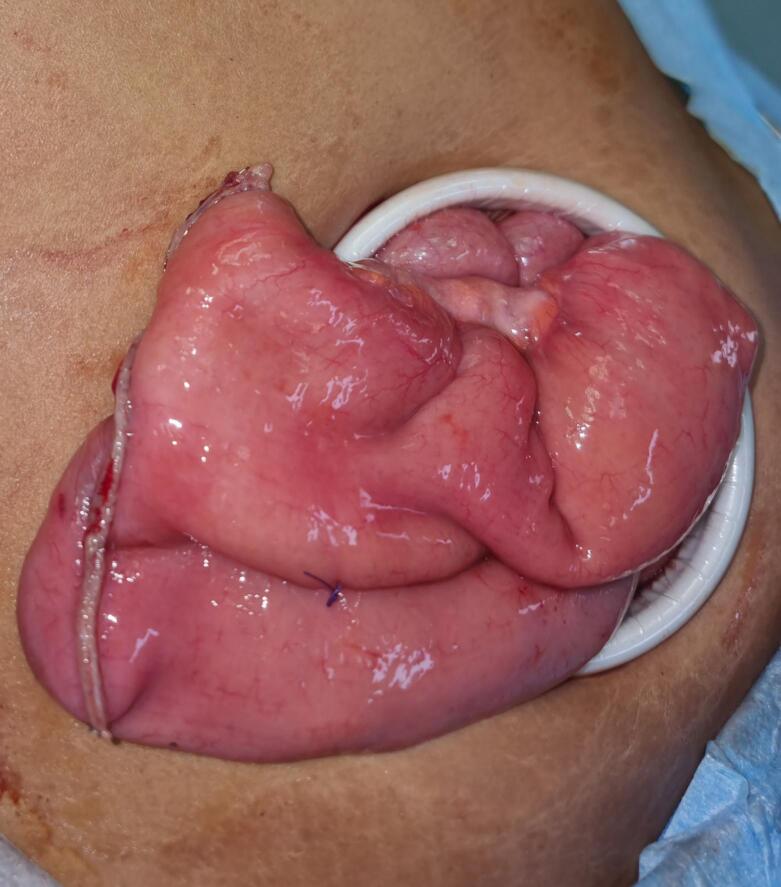
Extracorporeal end to end ileoileal anastomosis.

The patient underwent laparoscopic resection of a 10-cm ileal segment containing the polyp with side-to-side extracorporeal anastomosis (Fig. 3, Fig. 4). Gross examination of the surgical specimen revealed a 13-cm piece of small intestine with a pedunculated, well-circumscribed polypoid mass measuring 5 × 4.5 × 2 cm from the mucosal surface (Fig. 5). The polyp had a glistening smooth external surface with foci of focal congestion and superficial ulceration.

Intraoperative inspection localized the polypoid lesion approximately 100 cm proximal to the ileocolic anastomosis, remote from the typical location of a Meckel's diverticulum (reported mean distance 52.4 cm from the ileocecal valve) [11]. The lesion was strictly intraluminal and pedunculated with a narrow mucosal stalk; there was no serosal outpouching, no separate diverticular sac, and no independent mesenteric blood supply identified on the serosal surface.

**Fig. 6:**
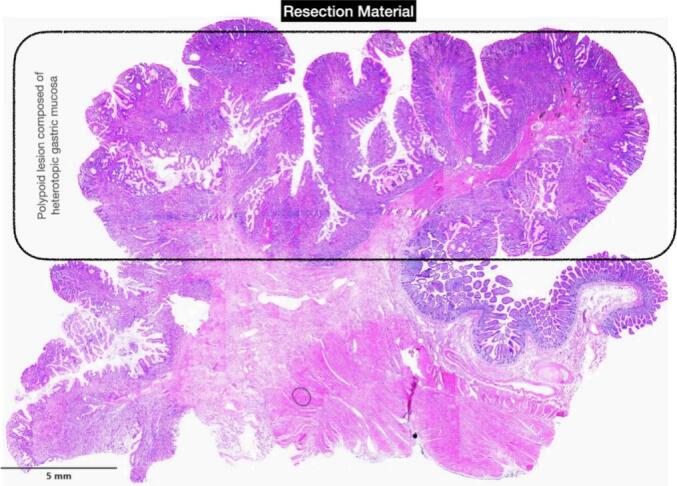
Higher view of the junction of heterotopic gastric mucosa and small intestinal mucosa, surgically resected specimen.

Histopathology described gastric-type epithelium in the ileal polyp, confirming the gastric origin of the tissue, without evidence of a full-thickness diverticular wall or duplication cyst. These macroscopic and microscopic features therefore support true mucosal heterotopia rather than a Meckel's diverticulum or an intestinal duplication. *Helicobacter pylori* infection, intestinal metaplasia, dysplasia, or malignancy was not observed. The covering ileal mucosa showed nonspecific inflammation with reactive lymphoid hyperplasia, which was very likely secondary to the intussusception process (Fig. 6).

The patient's postoperative course was uneventful. She was started with clear liquids on the second postoperative day and advanced to regular diet on the third day. Normalization of bowel function occurred on the third postoperative day with flatus passage, followed by normal bowel movements on the fourth day. She was discharged home on postoperative day five in good health with instructions regarding wound care and diet.

At two weeks, the patient reported complete resolution of symptoms and had resumed normal activities by six weeks. Given the rarity of this pathology and her history of recurrence, we recommended annual clinical follow-up with selective imaging for at least five years was recommended to monitor for recurrence (Table 1).

**Table 1:** Clinical course timeline of the patient.

Time point	Clinical event
7 years prior to presentation	Right ileocecectomy for intussusception with benign inflammatory as lead point
3 months prior to presentation	Onset of intermittent mild abdominal pain, self-resolving
Day 0	Presentation to surgical department with 6-day history of worsening colicky abdominal pain
Day 1	Abdominal ultrasonography and CT scan revealing ileoileal intussusception
Day 2	Colonoscopy with successful reduction of intussusception and identification of ileal polyp; biopsies obtained
Day 4	Biopsy results confirming gastric heterotopia
Day 5	Laparoscopic-assisted resection of ileal segment containing polyp with primary anastomosis
Day 10	Discharge from hospital
1-month post-surgery	follow-up with no evidence of recurrence

## 3 Discussion

This case serves as a reminder to consider uncommon etiologies such as gastric heterotopia as lead points for recurrent intussusception, even in the context of previous intestinal resection. In contrast to pediatric intussusception that is largely idiopathic (90 %), the lead point is identifiable in 70–90 % of the adult cases of intussusception [1]. The most common etiology was neoplasms (63 %), followed by postoperative adhesions (18 %), inflammatory lesions (8 %), and miscellaneous conditions (11 %) [12]. Pathological lead points vary in their distribution based on anatomical location, with malignancy being more common in colonic intussusception (62.5 %) than in small intestinal intussusception (16.7 %) [12].

Differential diagnoses for an ileal polypoid lesion include hamartomatous polyps conventional adenoma, submucosal tumors such as gastrointestinal stromal tumor or lipoma, duplication cyst or Meckel's diverticulum, and inflammatory or iatrogenic polyps [13]. In our patient, cross-sectional imaging and endoscopy demonstrated a discrete, intraluminal, pedunculated lesion (making a large subserosal or mesenteric mass unlikely, endoscopic biopsies revealed gastric-type epithelium rather than dysplastic glandular or spindle-cell morphology, and surgical/pathology inspection found no true diverticular wall or serosal outpouching to suggest Meckel's diverticulum or duplication [11]. Together these findings supported a diagnosis of isolated gastric mucosal heterotopia rather than the other entities above and informed a limited resection.

Heterotopic gastric mucosa is more commonly encountered in Meckel's diverticulum (50–60 % of cases), duodenum, and esophagus [4]. Histologically, heterotopic gastric tissue can contain any element of normal gastric mucosa, including oxyntic cells, chief cells, and mucus-secreting cells, with the potential to secrete acid and digestive enzymes [14]. The secretion can result in local inflammation, ulceration, bleeding, and, rarely, perforation [15].

The development of gastric heterotopia, defined as the presence of ectopic gastric mucosa outside its normal anatomical location, is multifactorial and still under investigation. One accepted theory suggests that it arises due to errors in the differentiation of pluripotent endodermal stem cells during the 4th to 7th weeks of gestation, leading to misplaced gastric tissue along the gastrointestinal tract [16]. This is supported by Pancreatic and Gastric Heterotopia in the Gallbladder [17], which explains that heterotopic pancreatic tissue is presumed to result from errors during embryological development. Another theory attributes it to a failure in the descent and proper positioning of the proximal intestine during embryogenesis [16].

In addition, anti-inflammatory strategies may limit inappropriate differentiation to avascular tissue. Z. Mungan et al. [18] showed the association between chronic inflammation and metaplastic changes in the GI tract, and hypothesized that targeted anti-inflammatory treatment could lessen the development of heterotopia. The clinical picture of adult intussusception is typically nonspecific and chronic [19,20]. The clinical presentation of adult intussusception varies widely, from acute abdomen to chronic, intermittent abdominal pain, as observed in our patient [21]. The duration of symptoms tends to be longer in adults than in children, with a mean of 37.8 days (range: 1–365 days) before diagnosis [1].

Imaging plays a significant role in diagnosis. Amr et al. [22] discussed the radiographic patterns of intussusception and stated that computed tomography (CT) was the most reliable in diagnosis, with sensitivity of 58 % to 100 % and specificity of 57 % to 71 %. Classic CT findings include the “target sign” (concentric rings of different density) or “sausage-shaped” configuration (alternating areas of low and high attenuation) [23,24]. Ultrasonography may reveal the classic “doughnut sign” or “pseudokidney sign” [25]. Also, colonoscopy allows the advantage of direct visualization, potential reduction of the intussusception, and biopsy of any lesion discovered [26].

The combination of gastric heterotopia causing recurrent intussusception after previous intestinal resection appears to be very rare (Table 2). Cai et al. [14] reported a case of ileal gastric heterotopia causing obstruction in a child with recurrent symptoms after initial resection, and B. WALTER and R. Schaefer [5] described a case of gastric heterotopic polyp in the rectum causing recurrent intussusception in an adult. Management of adult intussusception remains somewhat debated, particularly regarding the extent of resection and whether reduction should be attempted prior to resection. Azar and Berger [3] advocated for en bloc resection without reduction due to the potential for malignancy and the theoretical risk of tumor seeding or perforation with manipulation. A review of adult intussusception found that while the majority of cases require surgical intervention, the extent of resection should be individualized based on the underlying pathology [24].

**Table 2:** Clinical characteristics, surgical approaches, and outcomes of patients with recurrent intussusception caused by Ileal gastric heterotopia.

Authors (Year)	Patient population	Lead point identified	Diagnostic methods	Surgical approach	Recurrence after first surgery	Outcome	Clinical presentation	Size of heterotopic tissue
Eghbali et al. (2025)	31-year-old female	Ileal gastric heterotopia	Ultrasound, CT scan, colonoscopy	Laparoscopic resection	Yes	Full recovery	Abdominal pain	35 × 4.5 × 2 cm
Dourado, Fischer (2021) 1	6-year-old male	Ileal gastric heterotopia	Meckel's scan, MRI, ultrasound, laparotomy	Laparotomy with ileocolonic anastomosis	No	Full recovery	Abdominal pain, vomiting	Not specified
Anand et al. (2017) 2	6-year-old male	Ileal gastric heterotopia	Ultrasound, contrast enema	Surgical resection of affected ileal segment	No	Full recovery	Abdominal pain, intermittent melena	3 × 1.5 cm
Di Renzo, Rizzo, Fusillo (2019) 3	4-year-old male	Submucosal gastric heterotopia	Ultrasound, 99mTc pertechnetate scan, laparoscopy	Ileal resection	Yes	Full recovery	Abdominal pain, vomiting, failure to thrive	9 mm
Doberneck et al. (1976) 4	10-year-old male	Ileal gastric heterotopia	Meckel's scan, exploratory laparotomy	Ileal resection and anastomosis	No	Full recovery	Abdominal pain, vomiting	Not specified
Elemen et al. (2009) 5	4-year-old male	Ileal gastric heterotopia	Ultrasound, laparotomy	Ileal resection and anastomosis	Yes	Full recovery	Abdominal pain, intermittent vomiting	Not specified
Murshed et al. (2020) 6	33-year-old male	Ileal gastric heterotopia	CT scan, laparotomy	Ileal resection and side-to-side ileo-ileal anastomosis	No	Full recovery	Abdominal pain, nausea	4 cm
Cai, Yu (2017) 7	15-year-old male	Ileal gastric heterotopia	Capsule endoscopy, CECT, laparotomy	Ileal resection and anastomosis	No	Full recovery	Melena, abdominal pain	2.2 × 1.5 × 1.2 cm
Erez et al. (1991) 8	3-month-old infant	Ileal gastric heterotopia	Ultrasound, barium enema, laparotomy	Ileal resection and anastomosis	No	Full recovery	Abdominal pain, vomiting	1 cm
Wu et al. (2024) 9	19-year-old female	Ileal gastric heterotopia	CT scan, histopathology	Open right hemicolectomy, ileocolic anastomosis	No	Full recovery	Abdominal pain, nausea, vomiting	Not specified

1 Dourado JC, Fischer A: Small bowel heterotopic gastric mucosa as a lead point for recurring intussusception. *Journal of Pediatric Surgery Case Reports* 2021, 72:101946.

2 Anand P, Singh S, Sarin N: Intussusception caused by heterotopic gastric mucosa in small intestine: a case report. *Journal of medical case reports* 2017, 11:1–4.

3 Di Renzo D, Rizzo R, Fusillo M, Travascio L, Persico A, Lelli Chiesa P: Recurrent intussusception caused by submucosal, heterotopic gastric mucosa in the terminal ileum. *Journal of Pediatric Surgery Case Reports* 2019, 42:38–41.

4 Doberneck RC, Deane WM, Antoine JE: Ectopic gastric mucosa in the ileum: a cause of intussusception. *J Pediatr Surg* 1976, 11(1):99–100.

5 Elemen L, Oz F, Erdogan E: Heterotopic gastric mucosa leading to recurrent intussusceptions: report of a case. *Surgery today* 2009, 39(5):444–447.

6 Murshed KA, Khawar M, Petkar M: Heterotopic gastric mucosa in the ileum: a rare cause for intussusception in adults. *Case Reports in Gastroenterology* 2021, 14(3):609–614.

7 Cai J, Yu H: Giant polypoid gastric heterotopia in the small intestine in a boy: A case report and literature review. *Medicine* 2017, 96(1):e5854.

8 Erez I, Kovalivker M, Lew S, Lazar L, Motovic A: Ectopic gastric mucosa in a polyp causing ileo-ileal intussusception: a case report of a three-month-old baby. *European journal of pediatric surgery* 1991, 1(02):118–120.

9 Wu Y, Garza M, Russell E, Burch R, Wong M: S4936 Gastric Heterotopia Causing Intussusception in a Young Adult. *Official journal of the American College of Gastroenterology | ACG* 2024, 119(10S).

Surgery remains the gold standard for adult intussusception when a pathological lead point is identified. However, management differs by location; colonic intussusceptions are more often malignant and usually treated with en-bloc resection, whereas enteric intussusceptions are benign in the majority of cases and malignant tumors are found in only approximately 20 % of enteric cases [8,27]. A systematic review and meta-analysis also highlights that a substantial proportion of adult intussusceptions involve the small bowel and that outcomes are excellent when the pathological lead point is definitively removed [12]. Recent reports emphasise individualized decision-making for selected small-bowel lesions with reassuring imaging/endoscopic features, controlled reduction to permit targeted biopsy and a limited resection may be appropriate, while suspected malignant or colonic lesions are best resected en-bloc. Jha et al. [28] further illustrate the variable clinical presentations of adult intussusception and support tailoring the operative strategy to the clinical, radiologic and endoscopic context. Finally, when resection is required, minimally invasive techniques are increasingly used and offer advantages of reduced invasiveness, faster recovery and fewer wound complications [29].

As stated in the Case Presentation, we recommended annual clinical review for five years. Because formal surveillance guidelines for resected isolated ileal gastric heterotopia are lacking, this pragmatic institutional plan balances the low but uncertain recurrence risk against the harms of repeated ionising imaging. We recommend magnetic resonance (MR)-enterography as the preferred modality (baseline at 12 months with repeat at 36 months) and symptom-driven MR or capsule endoscopy earlier if concerns arise, with CT-enterography reserved only when MR is unavailable or urgent assessment is required.

Our case of recurrent intussusception due to ileal gastric heterotopia in a patient with previous ileocecectomy is an extremely rare clinical presentation and is consistent with the limited literature on this subject. Our approach to management of laparoscopic resection of the involved intestinal segment with primary anastomosis is in line with current guidelines for adult intussusception from benign lead points. Future studies should aim to develop standardized management algorithms for adult intussusception based on site, disease, and patient factors to improve outcomes in these rare conditions.

## 4 Conclusion

Ileal gastric heterotopia is a rare cause of recurrent adult intussusception after previous intestinal resection. Preoperative CT and endoscopy facilitate diagnosis and operative planning. For benign small-bowel lead points, limited segmental resection is definitive and yields favorable outcomes; when malignancy is suspected, en bloc resection is recommended.

## Abbreviations

CT: Computed Tomography
RLQ: Right Lower Quadrant
SCARE: Surgical Case Report
GI: Gastrointestinal
MR: Magnetic resonance

## Patient consent

Written informed consent was obtained from the patient for publication of this case report and accompanying images. A copy of the written consent is available for review by the Editor-in-Chief of journal upon request.

## Ethical approval

Ethical approval by the Research Committee was not necessary, as the format of this article is a case report.

## Funding

No funding was received for this case report.

## Author contribution

Writing – original draft: Dr Foolad Eghbali and Dr Mohammadsadra Shamohammadi.

Project administration: Dr Foolad Eghbali and Dr Mohammadsadra Shamohammadi.

Data Collection: Dr Nasir Mohseni jam, Dr Parisa Pooyan, and Dr Asghar Arabhosseini.

Patient's doctor: Foolad Eghbali and Dr Mohammadreza Javaherian.

Review and editing, supervision: Dr Mohammadsadra Shamohammadi.

## Guarantor

Dr. Foolad Eghbali and Dr. Mohammadsadra Shamohammadi.

## Research registration number

Not applicable.

## Declaration of Generative AI and AI-assisted technologies in the writing process

We only use these technologies to improve readability and language of manuscript.

## Conflict of interest statement

The authors declare no conflicts of interest regarding the publication of this case report.

## References

[1] Marinis A., Yiallourou A., Samanides L., Dafnios N., Anastasopoulos G., Vassiliou I., Theodosopoulos T. (2009). Intussusception of the bowel in adults: a review. World J. Gastroenterol.: WJG.

[2] Wang N., Cui X.-Y., Liu Y., Long J., Xu Y.-H., Guo R.-X., Guo K.-J. (2009). Adult intussusception: a retrospective review of 41 cases. World J. Gastroenterol.: WJG.

[3] Azar T., Berger D.L. (1997). Adult intussusception. Ann. Surg..

[4] Ali S.M., Ahmed A.A., Saaid L.A.H., Mohamed G.M.K., Shah A.A., Al-Tarakji M., Aftab Z. (2019). Inamulla, Rashid S: heterotopic gastric mucosa presenting as lower gastrointestinal bleeding: an unusual case report. Case Rep. Surg..

[5] B W.A.L.T.E.R., Schaefer R. (1961). Gastric heterotopia in the rectum: report of a case. Ann. Surg..

[6] Choi S.H., Han J.K., Kim S.H., Lee J.M., Lee K.H., Kim Y.J., An S.K., Choi B.I. (2004). Intussusception in adults: from stomach to rectum. Am. J. Roentgenol..

[7] Kerwan A., Al-Jabir A., Mathew G., Sohrabi C., Rashid R., Franchi T., Nicola M., Agha M., Agha R. (2025). Revised surgical CAse REport (SCARE) guideline: an update for the age of artificial intelligence. Premier J. Sci..

[8] Kouladouros K., Gärtner D., Münch S., Paul M., Schön M.R. (2015). Recurrent intussusception as initial manifestation of primary intestinal melanoma: case report and literature review. World J. Gastroenterol..

[9] Zubaidi A., Al-Saif F., Silverman R. (2006). Adult intussusception: a retrospective review. Dis. Colon Rectum.

[10] TC J., R R., Ganesh M.S. (2024). Adult intussusception: a systematic review of current literature. Langenbeck’s Arch. Surg..

[11] Hansen C.C., Søreide K. (2018). Systematic review of epidemiology, presentation, and management of Meckel’s diverticulum in the 21st century. Medicine (Baltimore).

[12] Hong K.D., Kim J., Ji W., Wexner S. (2019). Adult intussusception: a systematic review and meta-analysis. Tech. Coloproctol..

[13] Gore R.M., Mehta U.K., Berlin J.W., Rao V., Newmark G.M. (2006). Diagnosis and staging of small bowel tumours. Cancer Imaging.

[14] Cai J., Yu H. (2017). Giant polypoid gastric heterotopia in the small intestine in a boy: a case report and literature review. Medicine.

[15] Lupu V.V., Ignat A., Paduraru G., Mihaila D., Burlea M., Ciubara A. (2015). Heterotopic gastric mucosa in the distal part of esophagus in a teenager: case report. Medicine.

[16] Maconi G., Pace F., Vago L., Carsana L., Bargiggia S., Porro G.B. (2000). Prevalence and clinical features of heterotopic gastric mucosa in the upper oesophagus (inlet patch). Eur. J. Gastroenterol. Hepatol..

[17] Kantor M., Eiseler S., Schiller A., Hughes S., Liu X., Lai J. (2018). Pancreatic and gastric heterotopic tissue presenting as a symptomatic gallbladder mass: a case report and literature review. Clin. Res. Hepatol. Gastroenterol..

[18] Mungan Z. (2014). Is it Barrett’s esophagus or gastric heterotopia. Case Rep. Gastroenterol..

[19] Manjunath B.T., Reddy A.K., Krishnappa N., Shukla A.K. (2019). Adult intussusception: a case series. J. Med. Sci..

[20] Lu T., Y-m Chng (2015). Adult intussusception. Perm. J..

[21] Lianos G., Xeropotamos N., Bali C., Baltogiannis G., Ignatiadou E. (2013). Adult bowel intussusception: presentation, location, etiology, diagnosis and treatment. Il G. Chir.-J. Ital. Surg. Assoc..

[22] Amr M.A., Polites S.F., Alzghari M., Onkendi E.O., Grotz T.E., Zielinski M.D. (2015). Intussusception in adults and the role of evolving computed tomography technology. Am. J. Surg..

[23] Goi G., Guerci C., Ferrario L., Lamperti G.M.B., Cammarata F., Kazemi Nava A., Danelli P. (2025). Ileocolic intussusception: a case report and literature review. J. Surg. Case Rep..

[24] T. Chand J., R R., Ganesh M.S. (2024). Adult intussusception: a systematic review of current literature. Langenbeck’s Arch. Surg..

[25] Paulvannan V., Bylapudi S., Ramesh Kumar M.K., Nachimuthu M., Subramanian P. (2019). Perforation of heterotopic gastric mucosa in ileal duplication in an adult: a case report. J. Surg. Case Rep..

[26] Zhao G., Meng W., Bai L., Li Q. (2022). Case report: An adult intussusception caused by ascending colon cancer. Front. Surg..

[27] Marsicovetere P., Ivatury S.J., White B., Holubar S.D. (2017). Intestinal intussusception: etiology, diagnosis, and treatment. Clin. Colon Rectal Surg..

[28] Jha R., Ghimire S.K., Prasad R., Thapa K.S., Jha S., Singh P. (2025). Case report of a rare variant of adult intussusception with delayed presentation: idiopathic ileocecal colic intussusception. Ann. Med. Surg. (Lond)..

[29] Shenoy S. (2017). Adult intussusception: a case series and review. World J. Gastrointest. Endosc..

